# Critical Minireview: The Fate of tRNA^Cys^ during Oxidative Stress in *Bacillus subtilis*

**DOI:** 10.3390/biom7010006

**Published:** 2017-01-20

**Authors:** Juan Campos Guillen, George H. Jones, Carlos Saldaña Gutiérrez, José Luis Hernández-Flores, Julio Alfonso Cruz Medina, José Humberto Valenzuela Soto, Sergio Pacheco Hernández, Sergio Romero Gómez, Verónica Morales Tlalpan

**Affiliations:** 1 Facultad de Química, Universidad Autónoma de Querétaro, Cerro de las Campanas s/n, 76010 Querétaro, Qro., Mexico; jacm022003@yahoo.com.mx (J.A.C.M.); serpacheco18@gmail.com (S.P.H.); ser69rom@gmail.com (S.R.G.); 2 Department of Biology, Emory University, Atlanta, GA 30322, USA; ghjones@emory.edu; 3 Facultad de Ciencias Naturales, Universidad Autónoma de Querétaro, Cerro de las Campanas s/n, 76010 Querétaro, Qro., Mexico; doctorado.cs.uaq@gmail.com; 4 Centro de Investigación y de Estudios Avanzados del IPN, P.O. Box 629, 36500 Irapuato, GTO, Mexico; jhernand@ira.cinvestav.mx; 5 CONACYT, Centro de Investigación en Química Aplicada, Blvd. Enrique Reyna Hermosillo No. 140, 25294 Saltillo, Coahuila, Mexico; humberto.valenzuela@ciqa.edu.mx; 6 Facultad de Medicina, Universidad Autónoma de Querétaro, Cerro de las Campanas s/n, 76010 Querétaro, Qro., Mexico; vmtlalpan@gmail.com

**Keywords:** bacillithiol, *Bacillus*, oxidative stress, tRNA

## Abstract

Oxidative stress occurs when cells are exposed to elevated levels of reactive oxygen species that can damage biological molecules. One bacterial response to oxidative stress involves disulfide bond formation either between protein thiols or between protein thiols and low-molecular-weight (LMW) thiols. Bacillithiol was recently identified as a major low-molecular-weight thiol in *Bacillus subtilis* and related Firmicutes. Four genes (*bshA*, *bshB1*, *bshB2*, and *bshC*) are involved in bacillithiol biosynthesis. The *bshA* and *bshB1* genes are part of a seven-gene operon (*ypjD*), which includes the essential gene *cca*, encoding CCA-tRNA nucleotidyltransferase. The inclusion of *cca* in the operon containing bacillithiol biosynthetic genes suggests that the integrity of the 3′ terminus of tRNAs may also be important in oxidative stress. The addition of the 3′ terminal CCA sequence by CCA-tRNA nucleotidyltransferase to give rise to a mature tRNA and functional molecules ready for aminoacylation plays an essential role during translation and expression of the genetic code. Any defects in these processes, such as the accumulation of shorter and defective tRNAs under oxidative stress, might exert a deleterious effect on cells. This review summarizes the physiological link between tRNA^Cys^ regulation and oxidative stress in *Bacillus*.

## 1. Introduction

Redox reactions are essential to the metabolic economy of living systems. However, when cells are exposed to high concentrations of reactive oxygen species, numerous biochemical and physiological pathways may be affected, thus disrupting cellular homeostasis. For these reasons, organisms have developed strategies to limit the effects of oxidative stress on biological components. One of such strategies involves protein protection through thiol regulation. Disulfide bonds are important in a number of metabolic pathways, one of which is the interaction between proteins and low-molecular-weight (LMW) thiols during oxidative stress. Diverse prokaryotic and eukaryotic organisms have the ability to synthesize glutathione, bacillithiol, or mycothiol as part of their stress responses. These LMW thiols play an essential role in the maintenance of a reducing environment in the cytosol.

Another important cellular strategy involved in stress responses occurs at the level of tRNA metabolism, where tRNAs charged with their specific amino acids are essential for the biosynthesis of stress-related proteins. In addition, there is evidence to suggest that, when cells do not have the capacity to effectively regulate the levels of reactive oxygen species, pathways are induced that lead to tRNA degradation as a mechanism for regulating the expression of particular genes. Although the role of tRNAs in cellular metabolism is extensive, in this minireview, we will focus on the physiological link between bacillithiol biosynthesis and tRNA^Cys^ processing and regulation in *Bacillus subtilis*. Both molecules have molecular connections through the expression of genes from one operon (*ypjD*), which contains genes for both bacillithiol biosynthesis and tRNA maturation.

## 2. *Bacillus* under Oxidative Stress

*Bacillus* is a genus of Gram-positive bacteria, which are members of the family Bacillaceae. These bacteria are endospore formers and are obligate aerobes or facultative anaerobes [1,2]. Bacilli survive and disperse in the presence of other microorganisms in their natural environments. In the various environments in which they are found, Bacilli are subject to multiple sources of toxic oxidizing agents that induce internal production of reactive oxygen species [3]. Those conditions promote decisions between survival and cell death when damage to biomolecules is severe.

Under stress conditions, initiation signals result in the activation of the master transcription regulator, sigma factor σ^B^. Activated σ^B^ regulates the transcription of approximately 200 genes in response to multiple physical, chemical, and other environmental stress stimuli [4]. *Bacillus* cells contain robust mechanisms to respond to and mitigate environmental stress. These responses include the neutralization of stress stimuli by disulfide bonds, which play a major role in stabilizing protein structures or are part of their catalytic site. In addition, many proteins possess cysteine residues which function as redox switches, e.g., ribonucleotide reductase, RNase A, RNase T, methionine sulfoxide reductase, alkylhydroperoxide reductase, arsenate reductase, and the global repressor of the peroxide regulon PerR [5,6,7,8].

One important component of stress responses in *Bacillus* is the protection of exposed cysteine residues from fluctuations in the redox environment by bacillithiol (BSH), the α-anomeric glycoside of l-cysteinyl-d-glucosamine with l-malic acid. BSH is found in a wide range of bacteria including many Firmicutes [9].

## 3. Bacillithiol Biosynthesis and Function

**Figure 1:**
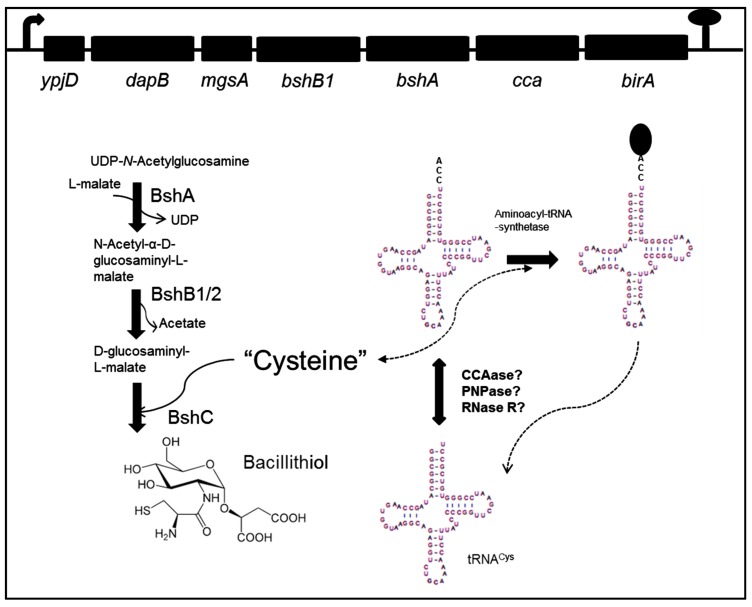
Possible relationship between tRNA^Cys^ and bacillithiol biosynthesis. Genes involved in bacillithiol biosynthesis are indicated in the operon ***ypjD***; *bshB1* (*N*-acetyl-α-d-glucosaminyl l-malate deacetylase) and *bshA* (*N*-acetyl-α-d-glucosaminyl l-malate synthase). The *bshB2* (*N*-acetyl-α-d-glucosaminyl l-malate deacetylase) gene is localized in the operon ***yoyC***, while ***bshC*** (d-glucosaminyl l-malate cysteine ligase) is situated in the operon ***ylbQ***. The *cca* (CCA, ATP (CTP): tRNA nucleotidyltransferase or CCAase) gene involved in tRNA maturation is indicated. A link between bacillithiol biosynthesis and tRNA^Cys^ maturation or degradation could have physiological relevance for *Bacillus subtilis* under stress conditions. Thus, the free cysteine pool would be necessary both for the synthesis of bacillithiol and for the aminoacylation of the single tRNA^Cys^ species in *B. subtilis*. The genes *ypjD* (pyrophosphohydrolase), *dapB* (dihydrodipicolinate reductase), *mgsA* (methylglyoxal synthase) and *birA* (biotin–protein ligase) are indicated in the operon. Polynucleotide phosphorylase (PNPase) and 3′-to-5′ processing exoribonuclease (RNase R) are indicated.

Three loci are involved in bacillithiol biosynthesis (*ypjD*, *yoyC,* and *ylbQ*), all of which are expressed from canonical σ^A^-dependent promoters. BSH biosynthesis (Figure 1) begins with the reaction of UDP-*N*-acetylglucosamine (UDP-GlcNAc) and l-malate to produce α-d-glucosaminyl l-malate (GlcNAc-Mal). This step is catalyzed by the enzyme BshA (*N*-acetyl-α-d-glucosaminyl l-malate synthase). The second step is deacetylation of GlcNAc-Mal to produce GlcN-Mal. This step is catalyzed by either of two redundant enzymes, BshB1 or BshB2 (*N*-acetyl-α-d-glucosaminyl l-malate deacetylase). The last step of BSH biosynthesis is achieved by coupling cysteine to GlcN-Mal, and is catalyzed by BshC (d-glucosaminyl l-malate cysteine ligase) [9,10]. BshA and BshC are essential in bacillithiol biosynthesis, while, in the absence of BshB1, bacillithiol production is reduced by up to 50%, indicative of the reduced activity of BshB2 [9,10].

The genes *bshA* and *bshB1*, whose products are involved in the first two steps of bacillithiol biosynthesis (Figure 1), are encoded in the same operon [9,10]. The operon begins with a putative pyrophosphohydrolase (*ypjD*), a dihydrodipicolinate reductase (*dapB*), and methylglyoxal synthase (*mgsA*). These are followed by the *bshB1* and *bshA* genes. Downstream of *bshA* are two genes, *cca* and *birA*, encoding CCA-tRNA nucleotidyltransferase and biotin–protein ligase, respectively [11,12,13].

Once bacillithiol is synthetized during stress conditions, it is responsible for reducing intra- or intermolecular disulfide bonds in cytosolic proteins. It does this by attacking disulfide bonds and forming diverse BSH-disulfide products. This mechanism maintains a reducing cytoplasmic environment that protects exposed cysteine residues from oxidation and reduces accumulation of proteins with non-functional disulfide bonds [7,14].

Despite the established role of BSH in the maintenance of disulfide homeostasis, there is evidence to suggest that it is not essential for activating oxidative and disulfide stress responses for some stress-related genes in *Bacillus subtilis.* For instance, Nakano et al. [14] used a 2D gel fluorescence-based thiol-modification assay to identify reversibly oxidized proteins during the induction of disulfide stress in *B. subtilis* with diamide [diazenedicarboxylic acid bis(*N*,*N*-dimethylamide)], a specific oxidant for thiols. Their analysis demonstrated that the protein redox status, including that of the Spx protein, a master regulator of disulfide (thioldepletion) stress [14], remained unchanged in wild-type, *bshA*, *bshB1*, and *bshB2* mutant strains [6,9]. Spx activity is controlled by a CXXC redox switch and, in its oxidized form, regulates transcription of target promoters, including those of the essential thioredoxin/thioredoxin reductase system encoded by *trxA* and *trxB*, and promoters for the operons *ypjD*, *yoyC,* and *ylbQ* encoding genes for bacillithiol synthesis [10]. Possible candidate molecules for the maintenance of protein redox status in the experiments just described are free cysteine and perhaps tRNA^Cys^ (see below). With regard to a possible role for free cysteine, some redundancy in the function of LMW thiols in *B. subtilis* is expected [6]. In fact, both BSH and cysteine could regulate the activity of OhrR, a cysteine-based peroxide sensor [15].

Despite the apparent redundancy in disulfide stress protection mechanisms in *B. subtilis*, BSH null cells do show striking changes compared with the wild type, e.g., reduced efficiency of sporulation, increased sensitivity to high concentrations of NaCl and low pH, and dramatically increased sensitivity to the antibiotic fosfomycin [9]. The broad-spectrum activity of fosfomycin against bacteria is due to its function as a potent covalent inhibitor of MurA, a key enzyme involved in peptidoglycan biosynthesis. MurA catalyzes the transfer of enolpyruvate from phosphoenolpyruvate to uridine diphospho-*N*-acetylglucosamine. The covalent inhibition of MurA results from a nucleophilic attack by an active-site cysteine thiol at the C-2 position of fosfomycin [16]. One way to inactivate fosfomycin is through fosfomycin-resistance enzymes such as FosA, FosB, and FosX. FosA is a manganese-dependent glutathione transferase identified in various Gram-negative bacteria. FosA catalyzes the reaction between fosfomycin and glutathione (GSH), leading to GSH dependent ring opening at the sterically hindered C-1 carbon of fosfomycin to form an inactive GSH–fosfomycin conjugate. FosB is a divalent-metal-dependent thiol-S-transferase related to FosA that has been identified in many low-G+C Gram-positive bacteria. FosB catalyzes the formation of an inactive complex between BSH and fosfomycin by nucleophilic attack of BSH at the C-1 carbon of fosfomycin [17,18]. FosX is a metal-dependent hydrolase found in *Mesorhizobium loti* and *Listeria monocytogenes*, which catalyzes the hydrolysis of the antibiotic [16]. These activities are primarily responsible for conferring fosfomycin resistance on *Bacillus subtilis* and other Gram-positive bacteria.

## 4. Role of CCA-tRNA Nucleotidyltransferase

As is shown in Figure 1, *cca*, the gene encoding CCA, ATP (CTP): tRNA nucleotidyltransferase (CCAase), is a component of the operon that contains *bshA* and *bshB1*. The principal function of CCAase is the addition of CCA residues to the 3′ end of tRNAs and tRNA-like transcripts, during maturation or recycling of these molecules. tRNAs are synthesized as precursors that undergo a series of post-transcriptional modifications, such as 5′ and 3′ processing. All mature tRNA molecules possess a functional CCA sequence at the 3′ end, as this sequence is required for amino acid attachment. Thus, CCAase activity is necessary to maintain levels of mature tRNAs ready to be used in protein synthesis and other biological reactions [19,20,21].

Experimental evidence has confirmed that all three loci involved in BSH biosynthesis are induced approximately fivefold during oxidative stress in *B. subtilis*, under control of the master regulator of disulfide (thioldepletion) stress, Spx [10]. This observation suggests the possibility that CCAase is also over-expressed in similar conditions and its activity could be necessary to maintain mature tRNA levels during stress. However, expression of *cca* and regulation of CCA addition at the 3′ of tRNA during stress conditions has not received much attention, presumably because of the assumption that damaged tRNA molecules that might be produced under such conditions are degraded efficiently by RNases.

With regard to the latter possibility, there is evidence indicating that CCAase has the ability to distinguish between normal and damaged tRNAs, e.g., tRNAs with poly(A) tails or with CCACCA sequences, for efficient CCA addition at the 3′ end. Such aberrant tRNAs are generally marked for degradation by RNases, as an important mechanism in successful quality control [22,23]. Indeed, the cleavage of tRNAs by specific RNases in response to multiple physical, chemical, and other environmental stresses has been detected in prokaryotes and eukaryotes and raises the possibility that this cleavage of tRNA, principally in the anticodon loop, could be a potentially important causative factor in the protection of cells from these stresses [24]. For instance, cleavage of tRNA^Lys^ in *Escherichia coli* by the PrrC nuclease, which is activated during bacteriophage T4 infection, has been reported [25]. Similarly, cleavage of tRNAs by RNase T2 and members of the RNase A family have been detected under stress conditions in yeast and mammalian cells [26,27]. tRNA cleavage during amino acid starvation in various biological systems has also been demonstrated [24]. All these reports indicate that tRNA cleavage is a common phenomenon in biological systems; its significance, regulation, and specific relationship to stress survival are unresolved questions.

## 5. A Link between Oxidative Stress, Bacillithiol, and tRNA^Cys^ Maturation?

In *Escherichia coli,* all tRNA genes encode the 3′ CCA [28]. In *B. subtilis,* on the other hand, around 30% of tRNAs lack an encoded CCA at the 3′ end (Table 1). CCA must be added post-transcriptionally to those CCA-less tRNAs [29,30].

The presence of *cca* in the *ypjD* operon, along with *bshA* and *bshB1* suggests the intriguing possibility that CCAase may be involved in the response of *B. subtilis* to oxidative stress. Evidence supporting this possibility was recently presented by Cruz Hernández et al. [29]. In that study, *B. subtilis* was subjected to oxidative stress generated by exposure to mercury, and the integrity of six tRNAs was then examined by electrophoretic mobility on Northern blots, finding that mercury exposure had no effect on the mobility of five of the six tRNAs, i.e., those for alanine, tryptophan, valine, leucine, and threonine. In contrast, northern blots revealed a shorter species of tRNA^Cys^ that migrated faster than the mature tRNA^Cys^.

The smaller tRNA^Cys^ species observed in conditions of oxidative stress had an electrophoretic mobility identical to that previously reported for tRNA^Cys^ lacking the CCA end, as assessed using synthetic CCA-less tRNA^Cys^ as a molecular marker [30]. It must be noted that the purification and sequencing of the smaller tRNA^Cys^ species were not performed in this study. However, the analysis of some fragments of tRNA^Cys^ obtained by reverse transcription polymerase chain reaction (RT-PCR) did demonstrate the presence of tRNA^Cys^ species that had been modified by the addition of poly(A) or heteropolymeric 3′ tails, suggesting that these were immature or damaged species, targeted for degradation [30]. Thus, it seems likely that the smaller tRNA produced by oxidative stress induced by mercury exposure is CCA-less tRNA^Cys^. After 4 h of exposure to mercury, the shorter tRNA^Cys^ species accounted for approximately 10% of total tRNA^Cys^ (mature tRNA^Cys^ plus the shorter species).

Cruz Hernández et al. [29] also examined the effects of oxidative stress on the accumulation of the shorter tRNA^Cys^ species in *B. subtilis* mutants lacking the ribonucleases polynucleotide phosphorylase (PNPase) and RNase R. They observed a significant increase in the relative amount of the shorter tRNA^Cys^ species in the *pnp* mutant and in the *pnp rnr* double mutant such that, by 6 h following exposure to mercury, the shorter tRNA^Cys^ species accounted for 70% of the total tRNA^Cys^ observed. The shorter tRNA^Cys^ species was not observed in the *rnr* single mutant, suggesting that PNPase may be involved in either the generation of mature tRNA^Cys^ via its polymerization activity or in the degradation of the CCA-less tRNA^Cys^ via phosphorolysis. This suggestion is supported by results obtained from studies in *E. coli*. Reuven et al. demonstrated the accumulation of shorter species of tRNA^Cys^, and other tRNA species, in mutants lacking CCAase, poly(A) polymerase, and PNPase [31].

Of additional interest was the observation by Cruz Hernandez et al. that the relative amount of the shorter tRNA^Cys^ species decreased after 6 h of oxidative stress [29]. By 10 h post-exposure to mercury, the relative level of the shorter species had decreased to less than 1% of the total in the wild-type strain and to ca. 10% of the total in the *pnp* mutant strain. The decrease in the relative amount of the putative CCA-less species with time of exposure to mercury can be rationalized via the operation of heavy metal detoxification mechanisms that are activated under conditions of heavy metal-induced oxidative stress. The failure to observe the shorter tRNA^Cys^ species in the *rnr* mutant remains unexplained.

How might tRNA maturation or degradation be affected by oxidative stress? The results obtained by Cruz Hernández et al. [29] demonstrate a clear role for PNPase in the maturation, repair, or degradation of tRNA^Cys^ during stress conditions, a role which has been verified in various species, from bacteria to humans [32,33,34,35]. However, the observation of the putative CCA-less tRNA^Cys^ species also suggests an important role for CCAase itself in the production of mature tRNA^Cys^ during oxidative stress. CCAase activity is presumably necessary to produce mature tRNAs that are competent to participate in protein synthesis, but the results of Cruz Hernández et al. also raise the interesting question of whether there is a relationship between *cca* expression and the expression of the genes involved in BSH synthesis, as proposed in Figure 1. It is also possible that expression of the genes involved in BSH biosynthesis affects the production of cysteine (Figure 1), increasing the activity of cysteine biosynthetic genes, which are controlled by different regulators, including CymR, CysL, and SpX [6,7]. This increase would make cysteine available for BSH synthesis and for the function of the free amino acid as an LMW thiol.

tRNAs are generally considered to be relatively stable molecules in cells because they have a complex secondary structure, contain protective nucleotide sequences at their 3′ ends and because they are associated with components of the translational machinery that reduce the action of RNases [36,37]. Nevertheless, there is evidence that supports global changes in the tRNA pool in different cellular states or under various growth conditions [38,39]. For instance, it has been shown in *E. coli* that starvation for a single amino acid results in reduced levels of the corresponding aminoacylated tRNA and degradation of this tRNA is accelerated during the first 20 minutes following the initiation of starvation [38,39]. Given these observations, we speculate that, during stress conditions in *B. subtilis*, cysteine depletion or other effects on components of the translation machinery, such as aminoacyl-tRNA synthetases, may increase uncharged tRNA^Cys^ accumulation. This situation could lead to an increased rate of degradation of the uncharged tRNA^Cys^ by various RNases, resulting in the buildup of CCA-less tRNA^Cys^ species. Thus, under these conditions, increased levels of CCAase may be necessary for CCA repair.

If such mechanisms are operative in *B. subtilis*, there must be some feature of them that limits their effects to tRNA^Cys^, at least among the tRNAs examined by Cruz Hernández et al. [29]. One possible reason for this specificity might involve the requirement for cysteine in the formation of bacillithiol and the possible link between tRNA^Cys^ and cysteine levels (S-thiolation). Further work will be necessary to explore these hypotheses.

## 6. Concluding Remarks

Available evidence suggests the interesting possibility that there may be a biologically significant relationship between the maturation or decay of tRNA^Cys^ and the response of *B. subtilis* to oxidative stress. This evidence suggests the further possibility that tRNA^Cys^ may play a role in the organism’s metabolic processes other than its role in translation. For example, tRNA^Cys^ might function as a store of cysteine for bacillithiol biosynthesis or might otherwise play a role in the protection of cysteine residues from oxidation in various stress-related proteins. The combination of these effects would lead to the rapid readjustment of aminoacylated tRNA^Cys^ levels, ensuring tRNA quality control to maintain cell integrity. These possibilities await experimental verification.

## Figures and Tables

**Table 1:** Distribution of tRNA isotypes with and without an encoded 3′ CCA in *B. subtilis*.

**Isotype**	**Ala**	**Gly**	**Pro**	**Thr**	**Val**	**Ser**	**Arg**	**Leu**	**Phe**	**Asn**
With CCA	4	7	3	2	4	5	4	2	2	3
Without CCA	2	0	0	3	1	0	3	6	1	1
**Isotype**	**Lys**	**Asp**	**Glu**	**His**	**Gln**	**Ile**	**Mel**	**Tyr**	**Cys**	**Trp**
With CCA	4	4	1	2	1	3	6	2	0	1
Without CCA	0	0	5	0	3	0	0	0	1	0
